# Simulation of huntington’s disease in forensic psychiatry: Case report

**DOI:** 10.1192/j.eurpsy.2021.1896

**Published:** 2021-08-13

**Authors:** R. Almeida Leite, A. Costa, T. Santos, M. Colón

**Affiliations:** 1 Psychiatry And Mental Health, Baixo Vouga Hospital Centre, Aveiro, Portugal; 2 Center Delegation, National Institute of Forensic Medicine and Sciences, Forensic Medical Office, Coimbra, Portugal

**Keywords:** Personality, forensic psychiatric, Huntington Disease, violence

## Abstract

**Introduction:**

Huntington Disease (HD) is an autosomal-dominant, neurodegenerative disorder, with a progressive course, that typically involves a triad of cognitive, motor and psychiatric disorders. Its pathogenic mechanisms are not fully understood, although a faultily encoded version of the protein huntingtin—resulting from a cytosine-adenine-guanine (CAG) trinucleotide expansion in the HTT gene—has been shown to cause intracellular toxicity in neural tissue. Patients usually presents with prodromic psychiatric perturbances, such as depression, delusions or personality changes. Occasionally HD gives rise to criminal behavior.

**Objectives:**

To understand HD clinical presentation and underlines the differencial diagnosis. We present a case of a 31-year-old male offender, whose mother was diagnosed with HD, and during his forensic-psychiatric evaluation, HD was considered, but not confirmed.

**Methods:**

Case report.

**Results:**

A 31-year-old male offender was under a forensic-psychiatric evaluation due to a crime of domestic violence, after he discovers that his wife had an affair. He reports previous personality changes and depression, and compares himself with his mother, stating she was diagnosed with HD due to psychiatric prodromic disturbances. He shows concern about having a disease, and was waiting for genetic test result. After a clinical evaluation, and despite a family history of HD and genetic suspicion, it was important to consider differential diagnosis. The case refers to a passionate crime, which attempted to simulate a HD, considering his genetic background.

**Conclusions:**

Psychometrically identifiable features in HD appear to be important in the context of analyzing circumstances occasioning criminal acts, but the medical history is the most important part of the examination.

**Disclosure:**

No significant relationships.

